# AMPK/SIRT1/PGC‐1α Signaling Pathway: Molecular Mechanisms and Targeted Strategies From Energy Homeostasis Regulation to Disease Therapy

**DOI:** 10.1111/cns.70657

**Published:** 2025-11-21

**Authors:** Junyang Chen, Boya Liu, Xinlei Yao, Xiaoming Yang, Jiacheng Sun, Jia Yi, Fei Xue, Jitai Zhang, Yuntian Shen, Bingqian Chen, Hualin Sun

**Affiliations:** ^1^ Jiangsu Key Laboratory of Tissue Engineering and Neuroregeneration, Key Laboratory of Neuroregeneration of Ministry of Education, Co‐Innovation Center of Neuroregeneration Nantong University Nantong Jiangsu Province China; ^2^ Department of Orthopedics Changshu Hospital Affiliated to Soochow University, First People's Hospital of Changshu City Changshu Jiangsu Province China

## Abstract

**Background:**

The AMPK/SIRT1/PGC‐1α pathway serves as a central regulator of cellular energy homeostasis, coordinating metabolic stress responses, epigenetic modifications, and transcriptional programs. Its dysfunction is implicated in the pathogenesis of a wide spectrum of complex modern diseases, spanning neurodegeneration, metabolic syndromes, and chronic inflammatory conditions. This review examines the pathway's role as an integrative hub and its potential as a therapeutic target.

**Methods:**

We synthesize current mechanistic evidence from molecular, cellular, and preclinical studies to elucidate the pathway's operational logic and the consequences of its dysregulation. The analysis is structured around key disease paradigms—including Alzheimer's disease, Parkinson's disease, diabetes, cardiovascular injury, stroke, and chronic kidney disease—to dissect its tissue‐specific pathophysiological impacts.

**Results:**

The AMPK/SIRT1/PGC‐1α axis operates through a core positive feedback loop: AMPK activation elevates NAD+, thereby activating SIRT1, which in turn deacetylates and activates PGC‐1α to drive mitochondrial biogenesis and function, further reinforcing SIRT1 activity. Disruption of this cascade manifests in disease‐specific mechanisms: promoting Aβ production via BACE1/γ‐secretase in Alzheimer's; impairing α‐synuclein clearance in Parkinson's; disrupting GLUT4 translocation and insulin signaling in diabetes; exacerbating oxidative damage and mitochondrial dysfunction in cardiovascular and neuronal injury; and accelerating fibrosis and sustained inflammation in renal and pulmonary diseases via NLRP3 and TGF‐β/Smad3 signaling.

**Conclusions:**

The AMPK/SIRT1/PGC‐1α pathway represents a cornerstone target at the intersection of metabolism, aging, and disease. Current therapeutic strategies—including pharmacological activators (e.g., metformin, SRT1720), natural compounds (e.g., resveratrol), lifestyle interventions (e.g., exercise, caloric restriction), and emerging technologies (e.g., gene editing, exosomal miRNAs)—offer multidimensional avenues for intervention. Future research must prioritize elucidating tissue‐specific regulatory mechanisms, such as AMPK isoform diversity and PGC‐1α interactome dynamics, to enable precision therapeutics and successful clinical translation for a range of complex disorders.

## Graphical abstract

The AMPK/SIRT1/PGC‐1α signaling axis integrates energy sensing, epigenetic regulation, and mitochondrial biogenesis to maintain cellular homeostasis. Dysregulation of this pathway is related to neurodegenerative, metabolic, and cardiovascular diseases, highlighting its potential as a multifaceted therapeutic target.

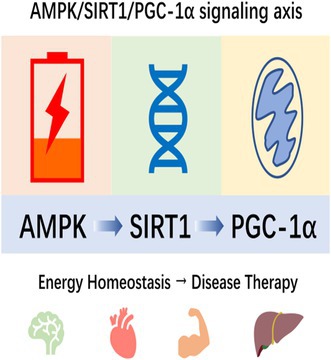

## 1 Introduction

Cellular energy homeostasis serves as the cornerstone of biological processes, governed by intricate signaling networks. Among these regulatory systems, the AMPK/SIRT1/PGC‐1α signaling axis emerges as a central hub for metabolic equilibrium by integrating energy sensing, epigenetic reprogramming, and transcriptional control. This pathway not only dynamically responds to energy fluctuations (e.g., changes in AMP/ATP ratios) but also orchestrates a sophisticated cascade‐feedback network through AMPK's metabolic switch function, SIRT1's senescence‐integrating capacity, and PGC‐1α's mitochondrial programming activity. Such coordination plays a pivotal role in cellular fate determination and organismal health maintenance. A deeper understanding of this pathway's regulatory mechanisms will offer novel therapeutic perspectives for addressing major public health challenges, including metabolic disorders, neurodegenerative diseases, and cardiovascular conditions.

As a master regulator of energy homeostasis, AMPK orchestrates physiological processes by integrating multidimensional metabolic signals. This enzymatic system responds to cellular energy fluctuations, reflected by changes in AMP/ATP and ADP/ATP ratios, and triggers a coordinated response that simultaneously boosts catabolic pathways for ATP generation while inhibiting non‐essential biosynthetic activities to restore energy balance [1, 2]. Intriguingly, AMPK exhibits context‐dependent roles in cancer, functioning as both a tumor suppressor and promoter through multilayered signaling cascades [3]. Moreover, as a key autophagy regulator, AMPK activation delays or even halts cellular senescence, highlighting its therapeutic potential in age‐related diseases such as cardiovascular disorders [4, 5]. Emerging evidence demonstrates that AMPK activation ameliorates metabolic dysfunction and other aging‐related phenotypes, whereas its inhibition impairs energy metabolism, mitochondrial biogenesis, and apoptosis [6, 7]. Collectively, AMPK functions as a cellular energy sensor that initiates dual metabolic reprogramming. Its functional versatility, evidenced by contrasting roles in both cancer progression and cytoprotection during aging, highlights its central importance in determining cell fate.

As an NAD^+^‐dependent deacetylase, SIRT1 orchestrates critical biological processes including energy metabolism, aging, and stress responses through dynamic modulation of substrate protein acetylation status [8]. This epigenetic regulator directly influences cellular fate decisions by deacetylating key proteins such as p53, thereby modulating cellular senescence and apoptosis [9]. During aging, the progressive decline in SIRT1 expression and activity contributes to cellular senescence phenotypes, while experimental enhancement of its activity has been shown to significantly attenuate aging processes and prolong cellular homeostasis [10]. These findings position SIRT1 as a promising therapeutic target, where mechanistic elucidation of its activation pathways and development of specific activators could not only delay physiological aging and maintain organismal health, but also provide novel treatment strategies for age‐related diseases including neurodegenerative and cardiovascular disorders. Importantly, SIRT1‐based anti‐aging interventions naturally extend to its downstream effectors (notably PGC‐1α)—a cascade that represents the central node for metabolic‐aging coordination in the AMPK/SIRT1/PGC‐1α axis.

PGC‐1α serves as a master transcriptional coactivator that governs energy metabolism, mitochondrial biogenesis, and functional adaptation [11, 12]. Through dynamic interactions with multiple transcription factors, it orchestrates gene expression programs that drive mitochondrial biogenesis, fatty acid oxidation, and adaptive thermogenesis [13]. Physiological challenges such as exercise or cold exposure induce PGC‐1α upregulation, which activates mitochondrial biogenic programs to enhance both mitochondrial quantity and quality, thereby elevating cellular energy metabolism [14, 15]. Concurrently, PGC‐1α modulates hepatic glucose homeostasis and skeletal muscle metabolism, playing indispensable roles in systemic energy balance [16]. As the terminal effector of the AMPK/SIRT1/PGC‐1α cascade, PGC‐1α embodies the final output of this signaling pathway by converting energy sensing through AMPK and epigenetic regulation via SIRT1 into transcriptional activation. This complete integration enables coordinated metabolic adaptation to diverse physiological demands.

The AMPK/SIRT1/PGC‐1α axis constitutes an evolutionarily conserved regulatory framework for maintaining cellular energy homeostasis, orchestrating a sophisticated signaling cascade that progresses from AMPK‐mediated energy sensing to SIRT1‐dependent epigenetic reprogramming, ultimately converging on PGC‐1α‐directed transcriptional control of metabolic programs. The pathway operates through a sophisticated feedforward‐feedback mechanism in which AMPK‐mediated phosphorylation of nicotinamide phosphoribosyltransferase (NAMPT) boosts intracellular NAD^+^ concentrations, resulting in subsequent SIRT1 activation [17]. Activated SIRT1 subsequently deacetylates PGC‐1α, enhancing its transcriptional activity to drive mitochondrial biogenesis and metabolic gene expression [18]. Completing this regulatory circuit, PGC‐1α reinforces SIRT1 expression and activity through a positive feedback loop, establishing a self‐amplifying metabolic control system [19]. This review systematically examines the pathway's regulatory mechanisms through three integrated dimensions, including molecular interactions, physiological responses, and pathological implications. Our synthesis provides a novel theoretical framework and targeted intervention strategies for major diseases including metabolic disorders and neurodegenerative conditions.

## 2 The AMPK/SIRT1/PGC‐1α Signaling Pathway: A Molecular Hub for Energy Homeostasis Regulation

### 2.1 AMPK: The Primary Sensor of Energy Stress

#### 2.1.1 The Multidimensional Regulatory Network of AMPK

AMPK functions as a heterotrimeric complex composed of α, β, and γ subunits, each contributing distinct structural and functional roles. The catalytic α subunit houses the kinase domain responsible for substrate phosphorylation, while the β subunit mediates complex assembly. The γ subunit contains four tandem cystathionine β‐synthase (CBS) domains that function as energy‐sensing modules by binding AMP, ADP, and ATP [5]. This molecular architecture enables precise kinase activity regulation through dual mechanisms. Under energy‐depleted conditions (characterized by elevated AMP/ADP and reduced ATP), AMP/ADP binding to the γ subunit induces allosteric changes that expose the kinase active site; conversely, ATP competitively binds to the CBS domains during nutrient‐replete conditions, effectively suppressing kinase activation and preventing unnecessary energy expenditure [20]. AMPK activation requires phosphorylation at Thr172 on its α subunit, a tightly regulated process mediated by distinct upstream kinases responding to different stimuli. Under metabolic stress, LKB1 phosphorylates AMPK during energy deprivation [21]. Calcium signaling triggers activation through CaMKKβ when intracellular Ca^2+^ levels rise. Cysteine dioxygenase type 1 (Cdo1) enhances this process by bridging CaMKK2 to AMPK [22, 23]. During inflammatory or oxidative stress, TGFβ‐activated kinase 1 (TAK1) mediates phosphorylation [24]. This multilayered regulatory system allows AMPK to integrate diverse cellular stress signals, precisely control downstream metabolic pathways, and maintain sensitive energy homeostasis.

AMPK's functional states are dynamically regulated through a sophisticated network of post‐translational modifications (PTMs) that extend beyond its canonical Thr172 phosphorylation. Multiple phosphorylation sites enable precise regulatory control. AKT‐mediated phosphorylation at α‐Ser485/491 suppresses AMPK activity, whereas β‐Ser108 autophosphorylation enhances kinase function. Additionally, AMPK‐dependent phosphorylation of GSDME at Thr6 inhibits pyroptosis [25, 26, 27]. Acetylation modifications also play a key role, with SIRT1 activating AMPK through α subunit deacetylation [28]. Additionally, ubiquitin‐dependent regulation occurs through MG53‐mediated degradation of AMPKα in response to glucose levels [29]. This intricate PTM network reveals that AMPK activity integrates not just energy status but also coordinated covalent modifications, creating multiple potential intervention points for therapeutic targeting. The system's complexity offers diverse opportunities for pharmacological manipulation of AMPK in drug development.

AMPK activity is subject to sophisticated allosteric regulation by small‐molecule compounds and therapeutic agents, forming an integrated pharmacological control network. The direct AMPK activators MK‐8722 and MT 63–78 both bind to an allosteric site on the β subunit to induce conformational activation [30]. Similar to AMP, MT 63–78 can also inhibit protein phosphatase 2Cα from dephosphorylating the AMPK Thr172 site, thereby maintaining the phosphorylated state of AMPK [31]. Similarly, 5‐aminoimidazole‐4‐carboxamide riboside (AICAR) undergoes intracellular conversion to ZMP, an AMP mimetic that triggers AMPK activation through structural mimicry of AMP's allosteric effects [32, 33]. In contrast, dorsomorphin competitively inhibits AMPK by reversibly binding to the catalytic domain, effectively blocking AICAR‐induced phosphorylation [34]. Beyond direct modulators, AMPK can be activated indirectly by compounds that elevate cellular AMP levels or calcium concentrations, exemplified by metformin's inhibition of mitochondrial complex I leading to AMP/ADP accumulation and subsequent AMPK activation [35, 36]. These interconnected regulatory mechanisms collectively influence diverse cellular processes including metabolism, growth, autophagy, and inflammatory responses, demonstrating AMPK's central role as a metabolic sensor integrating multiple pharmacological inputs.

#### 2.1.2 Central Role of AMPK in Metabolic Regulation and Cellular Homeostasis

As the primary cellular energy sensor, AMPK orchestrates a multifaceted metabolic network to maintain energy equilibrium. In glucose metabolism, AMPK enhances cellular glucose uptake by facilitating GLUT4 translocation to the plasma membrane and simultaneously activating rate‐limiting glycolytic enzymes such as phosphofructokinase‐1 [37]. This regulatory mechanism is particularly crucial in skeletal muscle, where AMPK mediates exercise‐induced GLUT4 membrane translocation, synergizing with insulin signaling to regulate glucose internalization [38]. Concurrently, AMPK potently suppresses hepatic gluconeogenesis through inhibition of the transcriptional coactivator CRTC2 [39]. Notably, impaired AMPK activity in type 2 diabetes mellitus (T2DM) patients contributes to defective glucose uptake and fatty acid oxidation, resulting in hyperglycemia [1], positioning AMPK activation as a promising therapeutic strategy for T2DM via mitochondrial function improvement.

In lipid metabolism, AMPK exerts inhibitory control over fatty acid synthesis through phosphorylation‐mediated inactivation of acetyl‐CoA carboxylase (ACC), thereby preventing excessive lipid accumulation [40]. In the regulation of proteostasis and autophagy, AMPK displays bifunctional control. On one hand, it maintains energy balance by suppressing mTORC1‐dependent translational processes under nutrient stress [41]. On the other hand, it directly stimulates autophagic flux via targeted phosphorylation of core autophagy regulators including ULK1 and BECN1 [42]. Furthermore, AMPK maintains mitochondrial integrity by coordinating mitochondrial biogenesis, dynamics (fusion/fission), and mitophagy [43, 44]. Collectively, AMPK serves as a metabolic guardian through its coordinated regulation of four fundamental processes, including glucose uptake enhancement, lipid metabolism suppression, autophagy initiation, and mitochondrial optimization. This integrated regulatory network not only preserves cellular energy homeostasis but also establishes AMPK as a natural defense hub against metabolic disorders such as T2DM.

### 2.2 SIRT1: The Master Epigenetic Regulator

SIRT1, a core member of the sirtuin protein family, functions as a NAD^+^‐dependent deacetylase localized in both nuclear and cytoplasmic compartments. Its conserved catalytic domain mediates NAD^+^‐dependent deacetylation reactions, while the N‐terminal STAC‐binding domain and intrinsically disordered regions regulate substrate recognition and protein interactions [45]. SIRT1 coordinates diverse cellular functions through targeted deacetylation mechanisms, demonstrating multifaceted regulatory roles. Metabolically, it activates PPARα via deacetylation to promote fatty acid oxidation while reducing lipid accumulation [46], while simultaneously enhancing insulin signaling sensitivity to improve glucose uptake and utilization for glycemic control [47, 48]. In mitochondrial regulation, SIRT1 amplifies SIRT3's deacetylation activity to preserve mitochondrial structure and function [49], and prevents pathological hyperfusion to maintain membrane integrity while inhibiting superoxide‐induced apoptosis [50]. Through these multifaceted roles, SIRT1 emerges as a master metabolic integrator, orchestrating NAD^+^‐dependent energy homeostasis, reciprocal regulation of lipid and carbohydrate metabolism, and mitochondrial optimization via crosstalk with SIRT3. It thereby serves as a crucial nexus between nutrient sensing and metabolic reprogramming.

SIRT1 coordinates a comprehensive cellular defense system through multifaceted protective mechanisms. By deacetylating FoxO1/FoxO3 transcription factors, it enhances antioxidant enzyme production (SOD, CAT) to combat oxidative stress [51, 52] For DNA protection, SIRT1 directly participates in homologous recombination and stabilizes repair proteins like Ku70 through deacetylation to maintain genomic integrity [53, 54]. In apoptosis regulation, it demonstrates dual protective capacity by inhibiting pro‐apoptotic p53 while enhancing anti‐apoptotic Bcl‐2 activity [55]. Neural protection is achieved through NLRP3 inflammasome suppression, preventing pathological cell death [56]. This comprehensive protective system integrates oxidative defense, genomic maintenance, and cell survival mechanisms, highlighting SIRT1's pivotal role as a stress response coordinator that connects epigenetic control with cellular protection pathways.

Emerging as a master regulator of longevity, SIRT1 orchestrates multiple anti‐aging pathways through its NAD^+^‐dependent deacetylase activity. At the genomic level, SIRT1 safeguards chromosomal integrity by deacetylating both histones and DNA repair proteins, enhancing various DNA damage repair pathways to minimize mutation accumulation and delay cellular senescence [10]. Its crucial role in stem cell maintenance is evidenced by studies showing that resveratrol‐mediated SIRT1 activation expands adult stem cell populations and extends lifespan in progeroid mouse models [57], while SIRT1 deficiency in hematopoietic stem cells accelerates aging phenotypes, leading to anemia and immunodeficiency [58]. Furthermore, SIRT1 exerts potent anti‐inflammatory effects by suppressing NF‐κB signaling and other pro‐inflammatory pathways, thereby attenuating chronic low‐grade inflammation (“inflammaging”) that characterizes the aging process [59, 60]. This comprehensive protective system, involving genomic stability maintenance, stem cell population conservation, and inflammasome modulation, elevates SIRT1 from a simple aging suppressor to a potential therapeutic template targeting fundamental senescence mechanisms.

Collectively, SIRT1 serves as a pivotal molecular regulator that integrates metabolic reprogramming, stress response pathways, and core aging hallmarks through its NAD + ‐dependent deacetylase activity. This multifaceted regulatory capacity establishes SIRT1 not only as a critical determinant of cellular rejuvenation but also as a validated molecular target for developing comprehensive anti‐aging strategies. The current understanding of SIRT1‐mediated longevity mechanisms provides a robust theoretical framework for designing multidimensional interventions targeting fundamental aging processes. Future investigations should prioritize the development of tissue‐specific delivery systems to enhance therapeutic precision, the optimization of temporal activation protocols to mimic physiological regulation, and the implementation of combinatorial approaches to address the complex nature of aging. These translational efforts will be essential for bridging the gap between mechanistic discoveries and clinically applicable anti‐aging interventions, ultimately facilitating the development of evidence‐based longevity medicine.

### 2.3 PGC‐1α: The Master Regulator of Mitochondrial Programming

Peroxisome proliferator‐activated receptor γ coactivator 1α (PGC‐1α) serves as the master regulator of mitochondrial biogenesis and energy metabolism, with its molecular architecture dictating its functional versatility [16]. Structurally, the protein integrates three specialized domains—an N‐terminal serine/arginine‐rich region mediating nuclear receptor engagement (PPARγ, ERRα), a central regulatory module harboring critical phosphorylation switches (Ser570, Thr262) that modulate protein turnover and functional output, and a C‐terminal RNA recognition motif (RRM) that orchestrates post‐transcriptional control through specific RNA interactions. As a prototypical transcriptional coactivator without DNA‐binding ability, PGC‐1α coordinates metabolic reprogramming via two complementary mechanisms. First, it facilitates transcriptional activation by recruiting histone acetyltransferases (CBP/p300) and chromatin remodeling complexes [61]. Second, it physically associates with nuclear respiratory factors (NRF1/NRF2) and mitochondrial transcription factor A (TFAM) to synchronize mitochondrial DNA replication, respiratory chain assembly, and oxidative phosphorylation [62]. This unique structural organization underlies PGC‐1α's tissue‐predominant expression in metabolically active organs (e.g., liver, muscle, brown adipose) [61, 63]. This selective distribution underlies its capacity to mediate distinct physiological adaptations in different tissues.

PGC‐1α orchestrates distinct metabolic programs across tissues through its unique DNA‐binding‐independent coactivation mechanism. During fasting adaptation in hepatocytes, it coordinates a dual metabolic response where HNF4α‐mediated transcriptional activation of gluconeogenic genes (PEPCK, G6Pase) maintains glucose production [64], while simultaneously enhancing fatty acid β‐oxidation to reduce hepatic lipid accumulation [65]. Skeletal muscle demonstrates PGC‐1α‐dependent fiber‐type plasticity, with overexpression promoting slow‐twitch fiber characteristics through MyHC I expression, mitochondrial biogenesis marked by cristae density increases, and enhanced oxidative capacity evidenced by elevated citrate synthase activity, collectively enhancing endurance performance [66, 67]. Brown adipose tissue utilizes PGC‐1α as a thermogenic switch, upregulating UCP1 and electron transport chain components to dissipate energy as heat during cold adaptation [68, 69]. In summary, PGC‐1α serves as a global orchestrator of cellular energy metabolism through its unique DNA‐binding‐domain‐independent coactivation mechanism and tissue‐specific regulatory networks. Its dynamic activity directly determines metabolic flexibility and stress adaptability, offering a critical therapeutic target for metabolic disorders and age‐related mitochondrial dysfunction.

## 3 The AMPK/SIRT1/PGC‐1α Signaling Cascade and Interaction Network

### 3.1 The Three‐Tiered Cascade Mechanism of the AMPK/SIRT1/PGC‐1α Signaling Pathway

**FIGURE 1:**
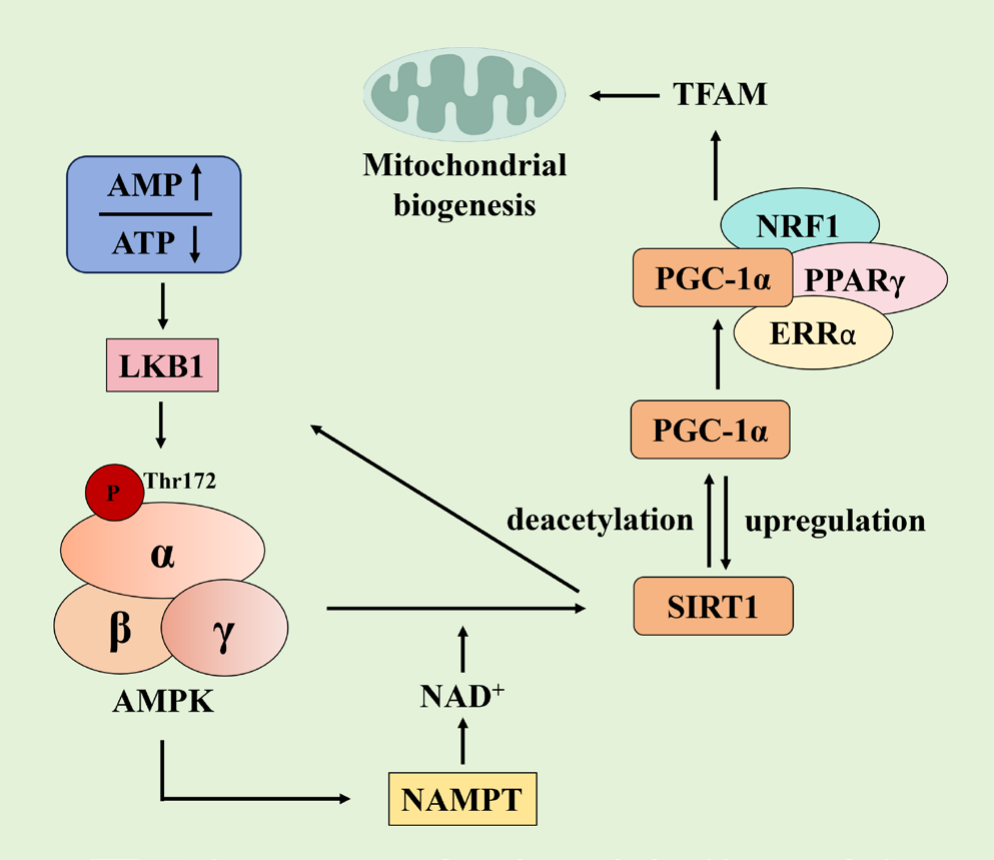
Energy Sensing to Mitochondrial Biogenesis. ATP decreases and AMP increases. AMP binds to the γ subunit of AMPK, inducing a conformational change that exposes Thr172 on the α subunit. LKB1 then phosphorylates Thr172, activating AMPK. AMPK directly phosphorylates NAMPT, thereby directly enhancing its activity. This leads to increased NAD^+^ levels. The elevated NAD^+^ levels activate the deacetylase activity of SIRT1. SIRT1 deacetylates PGC‐1α, increasing its transcriptional activity. PGC‐1α interacts with transcription factors such as PPARγ, ERRα, and NRF1 to upregulate TFAM expression. This promotes mitochondrial DNA replication and respiratory chain assembly, that is, mitochondrial biogenesis. SIRT1 can also deacetylate and activate LKB1, thereby enhancing AMPK activation. PGC‐1α, acting as a transcriptional coactivator, can upregulate SIRT1 expression.

The AMPK/SIRT1/PGC‐1α signaling pathway constitutes a sophisticated and finely tuned regulatory network, wherein key molecules exhibit intricate interactions and coordinated regulation to maintain cellular energy homeostasis and physiological functions (Figure 1). This pathway orchestrates energy balance through a three‐tiered cascade. Firstly, energy sensing and AMPK activation begin with declining ATP and rising AMP levels. These changes promote AMP binding to AMPK's γ subunit, inducing conformational changes that expose Thr172 on the α subunit. This exposure enables phosphorylation by kinases like LKB1, ultimately activating AMPK [20, 21]. Secondly, the NAD^+^‐SIRT1 axis is regulated through AMPK‐mediated phosphorylation and activation of NAMPT, the rate‐limiting enzyme in NAD^+^ biosynthesis. This process increases intracellular NAD^+^ levels, thereby enhancing SIRT1 deacetylase activity [70, 71]. Finally, mitochondrial biogenesis is enhanced through SIRT1‐mediated deacetylation of PGC‐1α, which increases its transcriptional activity. This modification enables PGC‐1α to interact with PPARγ/ERRα/NRF1 and upregulate TFAM expression, ultimately promoting mitochondrial DNA replication and respiratory chain assembly [72, 73]. Notably, AMPK, SIRT1, and PGC‐1α engage in intricate feedback mechanisms [74]. AMPK and SIRT1 mutually amplify each other's activity, while PGC‐1α forms a positive feedback loop by modulating SIRT1 expression and activity [75, 76]. Beyond indirect regulation via SIRT1, AMPK also directly phosphorylates PGC‐1α to enhance its stability and transcriptional activity [77]. In summary, AMPK senses AMP/ATP ratio changes, triggering Thr172 phosphorylation and activation. This upregulates the NAMPT‐NAD^+^ axis to potentiate SIRT1 deacetylation, which in turn modifies PGC‐1α to drive mitochondrial gene expression. The dynamic interplay among these components forms a self‐reinforcing network that balances energy metabolism and mitochondrial function. Elucidating this pathway's regulatory mechanisms advances our understanding of energy‐related diseases and provides critical targets for therapeutic development.

### 3.2 Cross‐Talk Between AMPK/SIRT1/PGC‐1α and Other Signaling Pathways

**FIGURE 2:**
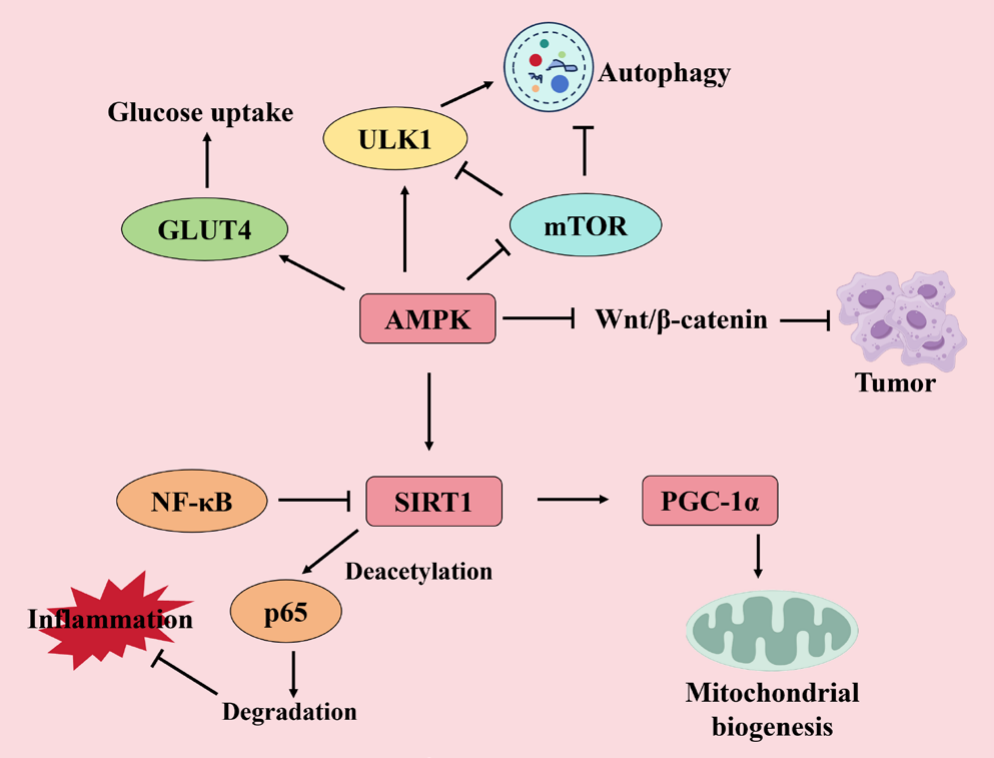
Cross‐talk with key signaling pathways in diseases. The central role of AMPK and SIRT1 in integrating metabolic signals (Glucose uptake, Mitochondrial biogenesis), autophagy processes (mTOR/ULK1), inflammation (NF‐κB), and tumor signaling (Wnt/β‐catenin). PGC‐1α is a key effector for mitochondrial function downstream of SIRT1/AMPK. Dysregulation of these interconnected pathways is important to many diseases.

The AMPK/SIRT1/PGC‐1α pathway engages in extensive cross‐talk with other signaling cascades, forming a multi‐layered regulatory network that fine‐tunes metabolic homeostasis (Figure 2). AMPK activation enhances insulin sensitivity, optimizing regulation of glucose transporter 4 (GLUT4) to promote glucose uptake [78]. The antagonistic relationship between AMPK and PI3K/AKT signaling plays a vital role in cancer development [79]. In antagonizing the NF‐κB pathway, SIRT1 deacetylates the NF‐κB subunit p65, promoting its proteasomal degradation and suppressing pro‐inflammatory cytokine expression. Conversely, NF‐κB activation inhibits SIRT1/PGC‐1α signaling and drives aerobic glycolysis, particularly in cardiovascular diseases, where this interplay ameliorates metabolic dysfunction and oxidative stress while counteracting NF‐κB‐mediated inflammatory damage [80]. Regarding mTOR‐dependent autophagy regulation, mTOR suppression enhances AMPK phosphorylation and upregulates autophagy‐related genes (e.g., LC3/ULK1), establishing the AMPK‐mTOR‐ULK1 axis as a central autophagy control mechanism [80]. Additionally, the pathway intersects with Wnt/β‐catenin signaling; for instance, in colorectal cancer, AMPK phosphorylates ubiquitin‐specific peptidase 10 (USP10) to inhibit Wnt/β‐catenin signaling and impede tumor progression [81]. These intricate feedback mechanisms enable the AMPK/SIRT1/PGC‐1α pathway to dynamically and precisely modulate cellular metabolism in response to energy status and physiological demands, ensuring homeostatic balance.

## 4 Role of the AMPK/SIRT1/PGC‐1α Pathway in Disease Pathogenesis

The AMPK/SIRT1/PGC‐1α signaling axis serves as a central regulatory network for cellular energy sensing, metabolic homeostasis, and stress response. Dysregulation of this pathway has been implicated in the pathogenesis of multiple major diseases. Below, we systematically elucidate its critical regulatory roles and therapeutic potential in neurodegenerative disorders, metabolic diseases, cardiovascular conditions, cerebral ischemia–reperfusion injury, and chronic inflammatory diseases.

### 4.1 Neurodegenerative Diseases

#### 4.1.1 Alzheimer's Disease

Alzheimer's disease (AD) presents three defining pathological hallmarks including β‐amyloid (Aβ) plaque accumulation, neurofibrillary tangles formed by hyperphosphorylated Tau protein, and progressive neuronal loss with synaptic dysfunction. The AMPK/SIRT1/PGC‐1α pathway demonstrates therapeutic potential by operating through three complementary mechanisms [82]. In Aβ metabolism regulation, AMPK activation suppresses BACE1/γ‐secretase expression, inhibiting aberrant cleavage of amyloid precursor protein (APP) and reducing Aβ production [83], while the AMPK‐ULK1 axis enhances autophagic flux to promote Aβ lysosomal degradation [84], and PGC‐1α boosts microglial phagocytic capacity for accelerated Aβ clearance [83, 85] (Table 1). For tau pathology modulation, SIRT1‐mediated Tau deacetylation effectively prevents its hyperphosphorylation and subsequent neurofibrillary tangle formation [86, 87]. Regarding neuroimmune regulation and mitochondrial restoration, the pathway drives microglial polarization from pro‐inflammatory (M1) to anti‐inflammatory (M2) phenotypes, reducing neurotoxic cytokine release while enhancing cellular antioxidant defenses [88], and PGC‐1α‐mediated mitochondrial biogenesis restores synaptic plasticity by improving energy metabolism [89]. This innovative therapeutic strategy achieves comprehensive intervention by concurrently addressing Aβ clearance, Tau pathology, neuroimmune modulation, and mitochondrial restoration, thereby overcoming the constraints of traditional single‐target approaches in Alzheimer's disease. The AMPK/SIRT1/PGC‐1α axis therefore emerges as a transformative platform for developing disease‐modifying therapies.

**TABLE 1:** Dysregulation of AMPK/SIRT1/PGC‐1α Signaling and Therapeutic Targets in Disease.

Disease category	Disease	Pathogenic mechanism	Therapeutic target/Strategy
Neurological Disorders	Alzheimer's Disease	BACE1 was upregulated lead to Aβ accumulation	AMPK activation: Promotes autophagy (via ULK1) for Aβ clearance; inhibits BACE1 SIRT1 activation: Deacetylates Tau, preventing hyperphosphorylation PGC‐1α activation: Enhances mitochondrial biogenesis and microglial phagocytosis
	Parkinson's Disease	α‐Synuclein aggregation	AMPK activation: Enhances autophagy/lysosomal function for α‐synuclein clearance SIRT1 overexpression: Rescues mitochondrial function, reduces apoptosis PGC‐1α activation: Restores mitochondrial biogenesis
	Amyotrophic Lateral Sclerosis	Mutant SOD1 aggregation	AMPK modulation: Induces autophagy to clear mutant proteins; inhibits mTOR SIRT1 activation: Enhances antioxidant capacity and autophagy PGC‐1α activation: Enhances mitochondrial biogenesis; reduces ROS; modulates neuromuscular junctions
Metabolic Diseases	Diabetes	GLUT4 translocation impairment lead to glucose uptake deficiency	AMPK activators (e.g., Metformin): Promote GLUT4 translocation, inhibit hepatic gluconeogenesis SIRT1 activators: Enhance insulin sensitivity, provide organ protection PGC‐1α activation: Restores mitochondrial biogenesis in muscle; activates brown fat thermogenesis
	Obesity	Downregulation of SIRT1 expression in adipose tissue cause metabolic dysregulation	AMPK activation: Inhibits lipogenesis, promotes fatty acid oxidation SIRT1 activation/PGC‐1α deacetylation: Enhances mitochondrial biogenesis, fatty acid oxidation, and thermogenesis
Cardiovascular Diseases	Coronary Artery Disease	Plaque buildup, narrowed arteries	AMPK/SIRT1 pathway activation: Promotes endothelial cell survival; inhibits vascular smooth muscle cell calcification
	Myocardial Ischemia–Reperfusion Injury	ATP depletion during ischemia, Excessive ROS production during reperfusion	AMPK/SIRT1/PGC‐1α activation: Reprograms metabolism, combats oxidative stress, preserves mitochondrial quality control, inhibits detrimental autophagy
Cerebral Ischemic Injury	Hypoxic–Ischemic Encephalopathy	Oxidative stress, neuroinflammation, Blood–brain barrier disruption	AMPK/SIRT1/PGC‐1α activation: Enhances mitochondrial biogenesis, reduces ROS, suppresses inflammation
	Cerebral Ischemia–Reperfusion Injury	Oxidative stress, inflammation, mitochondrial dysfunction post‐blood flow restoration	AMPK activation: Modulates inflammation, induces cytoprotective autophagy, maintains mitochondrial homeostasis; PGC‐1α activation: PGC‐1α enhances mitochondrial capacity via NRF1/TFAM
Chronic Inflammatory Diseases	Chronic Kidney Disease	SIRT1 downregulation, NLRP3 inflammasome overactivation; AMPK dysfunction, enhanced TGF‐β/Smad3 signaling, fibrosis	SIRT1 activation: Suppresses NLRP3 inflammasome; AMPK activation: Inhibits TGF‐β/Smad3 fibrotic signaling; PGC‐1α activation: Restores mitochondrial function and metabolic equilibrium
	Chronic Obstructive Pulmonary Disease	Cigarette smoke, oxidative stress and sustained inflammation	SIRT1 activators (e.g., Resveratrol): Boost antioxidants, rebalance MMP‐9/TIMP‐1; AMPK activators (e.g., Quercetin): Inhibit pro‐inflammatory signaling

#### 4.1.2 Parkinson's Disease

Parkinson's disease (PD) is characterized by progressive loss of dopaminergic neurons in the substantia nigra, accumulation of α‐synuclein aggregates (Lewy bodies), and gliosis, with pathogenesis involving complex interactions among mitochondrial dysfunction, oxidative stress, protein homeostasis disruption, and neuroinflammation [90, 91]. The AMPK/SIRT1/PGC‐1α pathway plays a central role in this process, and its dysregulation contributes to PD progression. PD involves AMPK dysfunction characterized by reduced AMPK phosphorylation in the substantia nigra, impairing cellular stress responses, where AMPK activation exerts neuroprotection through direct phosphorylation of ULK1 and mTORC1 inhibition to enhance autophagy, along with TFEB activation to promote lysosomal biogenesis, facilitating α‐synuclein clearance and improving neuronal survival in MPTP models [92, 93, 94, 95]. SIRT1 suppression in PD leads to downregulated SIRT1 expression, resulting in impaired autophagy and protein aggregation, mitochondrial dysfunction with increased ROS production, and apoptotic pathway activation, while SIRT1 overexpression rescues mitochondrial function and reduces neuronal apoptosis [96, 97]. Additionally, PGC‐1α deficiency contributes to mitochondrial failure, as reduced PGC‐1α expression decreases mitochondrial biogenesis and lowers ATP synthesis, whereas PGC‐1α activation restores respiratory chain function by upregulating NRF1/TFAM, ameliorating motor deficits [93, 98]. Collectively, the AMPK/SIRT1/PGC‐1α axis integrates energy sensing (AMPK), proteostasis (SIRT1), and mitochondrial function (PGC‐1α), forming a core neuroprotective mechanism in PD. Targeted modulation of this pathway offers a promising strategy to counteract dopaminergic neurodegeneration.

Recent research has revealed two promising therapeutic strategies for Parkinson's disease targeting the AMPK/SIRT1/PGC‐1α pathway. First, TPP‐modified macrophage membrane‐coated Cu₂₋ₓSe‐curcumin nanoparticles (CSCCT) selectively activate SIRT1/PGC‐1α signaling in degenerating neurons, promoting mitochondrial biogenesis with demonstrated improvements in complex I activity and motor function [99]. Second, peptide agonist teaghrelin simultaneously activates both AMPK/SIRT1/PGC‐1α and ERK1/2 pathways, effectively protecting against MPP^+^‐induced neuronal apoptosis and enhancing neuronal survival [92]. These interventions exert neuroprotection through three synergistic mechanisms, including the enhanced lysosomal degradation of α‐synuclein aggregates, the suppression of ROS generation via SOD2 activation, and the restoration of mitochondrial respiratory function through increased ATP production (Table 1). These findings establish that targeted activation of the AMPK/SIRT1/PGC‐1α pathway represents a novel multidimensional therapeutic paradigm for PD, simultaneously addressing protein homeostasis maintenance, oxidative stress mitigation, and mitochondrial functional recovery.

#### 4.1.3 Amyotrophic Lateral Sclerosis (ALS)

Amyotrophic lateral sclerosis (ALS) is characterized by progressive motor neuron degeneration leading to muscle atrophy [100, 101, 102], with pathogenesis involving mitochondrial accumulation of mutant SOD1 protein [103], glutamate excitotoxicity‐induced Ca^2+^ overload and mitochondrial ROS burst [104], as well as protein homeostasis disruption and neuroinflammatory cascades [105, 106], where the AMPK/SIRT1/PGC‐1α pathway plays a dynamic regulatory role (Table 1). Despite paradoxical AMPK hyperactivation in ALS models, its phosphorylation levels are significantly reduced [107], with appropriate AMPK activation exerting neuroprotective effects through induction of autophagy to clear mutant proteins (reduced SOD1 aggregates) [108], phosphorylation of mTOR negative regulators to inhibit mTORC1 signaling and subsequent protein translation [109], and promotion of mitochondrial biogenesis and fatty acid metabolism to potentially slow disease progression [110]. The SIRT1 regulatory network influences cellular aging processes and modulates stress/inflammatory responses in neurodegeneration [111], exerting protective effects in ALS through FOXO/PGC‐1α deacetylation to enhance antioxidant capacity (increased SOD2 activity) and activation of autophagy pathways for misfolded protein clearance (improved aggregate removal) [112, 113], with SIRT1 overexpression shown to mitigate SOD1 mutation‐induced ALS pathology [112]. Given PGC‐1α's reduced expression in ALS patient muscle tissue [114], its activation demonstrates therapeutic potential by enhancing mitochondrial biogenesis (improved respiratory chain complex activity), reducing ROS toxicity (decreased superoxide levels), and modulating neuromuscular junction gene expression [115, 116]. The AMPK/SIRT1/PGC‐1α axis thus provides a multidimensional intervention strategy targeting core ALS pathologies including mutant protein clearance (SOD1 aggregates), oxidative damage control (ROS burst), and mitochondrial reprogramming (respiratory chain repair/neuromuscular junction maintenance), with these findings not only revealing a reversible window for motor neuron degeneration but also establishing the molecular foundation for a “neuro‐metabolic homeostasis remodeling” therapeutic paradigm in ALS treatment.

### 4.2 Metabolic Disorders

#### 4.2.1 Diabetes Mellitus

Diabetes mellitus presents two fundamental pathological characteristics including insulin resistance and β‐cell dysfunction [117, 118, 119]. The AMPK/SIRT1/PGC‐1α pathway forms a coordinated regulatory network that improves metabolic disorders through multiple mechanisms. AMPK activation boosts glucose uptake in skeletal muscle by promoting GLUT4 movement [120, 121] and reduces liver fat production while increasing fat breakdown through CPT1 activation [122] (Table 1). Drug‐induced AMPK activation shows a significant improvement in blood sugar control and insulin response in obese animal models [123]. SIRT1 provides organ protection by modifying FOXO1/PGC‐1α to strengthen antioxidant defenses [124], relieving nerve damage in diabetes [125], and reducing kidney scarring by blocking TGF‐β signals [126]. PGC‐1α counteracts its low expression in diabetic muscles by promoting mitochondrial biogenesis [127], activating thermogenesis in brown fat to reduce blood sugar and fat levels, and cooperating with PPARα to improve muscle glucose use while suppressing hepatic gluconeogenesis [128]. The pathway enables a multifaceted diabetes intervention by simultaneously improving insulin signaling (AMPK‐dependent), normalizing metabolic flux (SIRT1‐mediated), and restoring mitochondrial function (PGC‐1α‐driven). These discoveries highlight the AMPK/SIRT1/PGC‐1α system as a key controller of diabetes development, presenting new treatment possibilities that target insulin function, sugar processing, and fat regulation through precise pathway adjustments.

#### 4.2.2 Obesity

Obesity, primarily caused by chronic energy intake exceeding expenditure, leads to excessive fat accumulation [129]. The core pathogenesis involves disrupted energy homeostasis. When energy intake surpasses adipose tissue storage capacity, circulating lipids accumulate in non‐adipose tissues, inducing lipotoxicity and pathophysiological alterations [130]. AMPK serves as a crucial cellular energy sensor that activates during energy deprivation to restore metabolic balance through complex signaling pathways [131]. Notably, both obese patients and animal models exhibit reduced AMPK activity [132, 133]. High‐fat diet‐induced obesity suppresses AMPK phosphorylation and subsequent autophagy, correlating with depressive and anxiety‐like behaviors in mice [134, 135]. Concurrently, obesity suppresses SIRT1 expression and activity, impairing its deacetylation of downstream targets [136] (Table 1). Research demonstrates that SIRT1 overexpression upregulates PPARγ mRNA levels, enhances Akt protein expression/activity, while downregulating hepatic FAS and ChREBP expression, collectively improving insulin sensitivity and reducing hepatic lipogenesis [137, 138]. Furthermore, SIRT1‐mediated PGC‐1α deacetylation enhances its transcriptional activity, promoting mitochondrial biogenesis and fatty acid oxidation to increase energy expenditure and thermogenesis [139]. Thus, the dysregulation of AMPK and SIRT1 signaling in obesity not only disrupts metabolic homeostasis but also contributes to associated neurobehavioral and hepatic complications, highlighting their potential as therapeutic targets for metabolic and psychiatric disorders.

Comprehensive mechanistic studies using adipocyte and hepatocyte models reveal that AMPK activation potently inhibits lipogenesis while promoting fatty acid oxidation. Specifically, activated AMPK phosphorylates and inhibits ACC, reducing malonyl‐CoA production and fatty acid synthesis, while upregulating OCTN2 to facilitate mitochondrial fatty acid transport and oxidation [140]. AMPK further amplifies these effects by modulating SIRT1/PGC‐1α activity, enhancing mitochondrial function and energy metabolism [141]. In obese murine models, AMPK/SIRT1 pathway activation reduces adiposity, enhances thermogenesis, and suppresses inflammation, ameliorating diet‐induced metabolic dysfunction [142]. These findings underscore the central role of AMPK/SIRT1 signaling in coordinating lipid metabolism and mitochondrial function, offering a promising therapeutic avenue for obesity‐related metabolic disorders through targeted pathway activation.

### 4.3 Cardiovascular Diseases

#### 4.3.1 Coronary Artery Disease

Coronary artery disease (CAD) is the top cause of heart‐related deaths. It happens when heart arteries narrow or get blocked by plaque buildup (Table 1). This reduces blood flow to the heart muscle. It can lead to serious problems like chest pain and heart attacks [143]. Scientists now believe the AMPK/SIRT1/PGC‐1α pathway could help treat CAD. AMPK activation protects the heart in two main ways. First, it helps keep artery lining cells alive [144]. Second, it changes how ACC works, which affects artery hardening [145]. Recent studies further elucidate AMPK's multifaceted role in CAD. In a large animal model of chronic myocardial ischemia, the GLP‐1 agonist Semaglutide was shown to improve myocardial perfusion and performance, an effect associated with increased activation of the endothelial‐protective AMPK pathway and downstream eNOS [146]. Additionally, AMPK activation also directly inhibits cardiomyocyte pyroptosis, a pro‐inflammatory cell death process implicated in coronary microembolization (CME)‐induced injury, via the AMPK/SIRT1/NLRP3 signaling axis [147]. Both AMPK and SIRT1 levels drop in CAD patients. Raising these levels with medicine might help [148]. Notably, the AMPK/SIRT1 axis also plays a crucial role in modulating atherosclerotic plaque stability. In diabetic settings, activation of AMPK and SIRT1 has been shown to enhance macrophage autophagy and reduce plaque vulnerability via pathways involving RAGE/LKB1/AMPK1/SIRT1, thereby stabilizing advanced atherosclerotic lesions [149]. A recent study found that Semen Ziziphi Spinosae extract reduces heart cell death by activating this pathway [150]. This research suggests new ways to treat CAD. The focus would be on protecting blood vessels, controlling metabolism, and preventing cell death. More studies are needed to find the best treatments. Scientists also need to understand exactly how this pathway works. They must check long‐term results in patients. This approach could lead to better, more targeted care for CAD.

#### 4.3.2 Myocardial Ischemia–Reperfusion Injury

Myocardial ischemia–reperfusion injury (MIRI) occurs when blood flow returns after a heart blockage but causes additional damage. This leads to cell death, irregular heartbeats, and weakened heart function [151]. The injury occurs through two sequential phases. Initially, oxygen deprivation depletes cellular ATP stores and disrupts ionic homeostasis. Subsequently, reperfusion generates excessive ROS that oxidatively damage lipids, proteins, and nucleic acids [152, 153] (Table 1). The AMPK/SIRT1/PGC‐1α pathway helps protect against MIRI in multiple ways. First, it reprograms cellular metabolism and combats oxidative stress through AMPK activation, which inhibits detrimental autophagy while preserving essential mitochondrial quality control [154]. Studies show AMPK acts as an energy sensor, reducing cell death and energy waste [155]. Second, it shields mitochondria by boosting antioxidant activity, lowering toxic ROS, and improving energy production [156, 157, 158, 159]. Key benefits include reducing oxidative stress, restoring energy balance, protecting mitochondria, and preventing cell death. Together, these effects help heart cells survive and recover. Better understanding this pathway could lead to new treatments for MIRI.

### 4.4 Cerebral Ischemic Injury

#### 4.4.1 Hypoxic–Ischemic Brain Injury

The brain needs constant oxygen and glucose to function properly. Hypoxic–ischemic brain injury (HIE) occurs when blood flow and oxygen to the brain suddenly stop during birth. This injury causes multiple problems including energy failure, oxidative stress, blood–brain barrier damage, brain inflammation, nerve cell toxicity, and blood vessel injury (Table 1) [160, 161, 162]. While cooling therapy helps, its benefits are limited [163], so we need better treatments. Mitochondria play a key role in HIE because they produce energy for brain cells. Helping mitochondria grow and function better could be an effective treatment [164]. When oxygen levels drop, AMPK activates and turns on SIRT1/PGC‐1α signaling. PGC‐1α works with NRF1 to increase UCP2 and TFAM, which helps mitochondria stay healthy, protects brain cells from stress, lowers harmful ROS, and keeps metabolism stable [165]. Studies show AMPK activation helps by reducing scar tissue formation, improving mitochondrial cleanup, and preventing brain cell death [166, 167, 168]. SIRT1 activation also fights inflammation by affecting the Nrf2–NF‐κB pathway, which decreases harmful chemicals and saves brain cells [169, 170]. Since SIRT1 helps control PGC‐1α [171], boosting the SIRT1–PGC‐1α–TFAM pathway improves mitochondria, reduces oxidative stress, and prevents cell death in HIE [172]. The AMPK/SIRT1/PGC‐1α pathway controls multiple aspects of HIE damage. It helps maintain energy, strengthens mitochondria, reduces inflammation, and protects brain cells. Targeting this pathway could lead to better treatments that improve outcomes for babies with HIE.

#### 4.4.2 Cerebral Ischemia–Reperfusion Injury

Ischemic stroke is the most common type of stroke, causing brain damage when blood flow stops. Restoring blood flow is the main treatment, but paradoxically can also cause additional harm to brain cells—this is called cerebral ischemia–reperfusion injury (CIRI) [173, 174]. The AMPK/SIRT1/PGC‐1α pathway helps protect the brain during CIRI by maintaining energy balance, reducing oxidative damage, and preventing cell death (Table 1). Following CIRI, AMPK exerts neuroprotection through two primary mechanisms. It modulates inflammatory responses by altering immune cell activity and inducing cytoprotective autophagy [175, 176]. Additionally, AMPK maintains mitochondrial homeostasis by facilitating damaged organelle clearance and regulating fission processes, with AMPK inhibition exacerbating cell death by impairing these quality control mechanisms [177, 178, 179]. SIRT1 and PGC‐1α function cooperatively in downstream pathways. SIRT1 activates SIRT3 to mitigate cerebral injury, apoptosis, oxidative damage, and mitochondrial dysfunction [49, 180]. Meanwhile, PGC‐1α enhances mitochondrial capacity by regulating biogenesis and function through the NRF1/TFAM axis [181]. Together, this pathway helps reprogram cell metabolism, repair oxidative damage, and optimize mitochondria. Future research should study how this pathway changes over time, how it interacts with brain blood vessels, and how to develop comprehensive protection strategies. These studies may lead to more precise stroke treatments.

### 4.5 Chronic Inflammatory Diseases

#### 4.5.1 Chronic Kidney Disease (CKD)

Chronic kidney disease (CKD) is characterized by progressive renal fibrosis, oxidative stress, and sustained inflammation [117, 182, 183, 184], with disease progression primarily driven by dysfunction of the AMPK/SIRT1/PGC‐1α signaling axis [185]. The malfunctioning AMPK/SIRT1/PGC‐1α pathway worsens chronic kidney disease through three connected problems (Table 1). First, when SIRT1 levels drop, it overactivates the NLRP3 inflammasome, causing excessive IL‐1β/IL‐18 release that damages kidney tubes [186]. Studies confirm CKD patients with lower SIRT1 have higher inflammation markers [187]. Second, when AMPK doesn't work properly, it boosts TGF‐β/Smad3 signals that activate scar‐forming cells and deposit harmful proteins [188, 189]. Research shows mice lacking AMPKα2 develop worse scarring [186, 187], while inactive SIRT1 prevents Smad3 breakdown, making scarring worse [190]. Third, low PGC‐1α impairs mitochondria's ability to produce energy in kidney cells, with CKD patients showing lower energy levels and more oxidative stress [191, 192]. Therapeutic modulation of this pathway yields multifaceted protection. SIRT1 exerts anti‐inflammatory effects via NLRP3 inflammasome suppression, with AMPK/SIRT1 cooperation inhibiting fibrotic progression through TGF‐β pathway regulation. Concurrently, PGC‐1α mediates mitochondrial rehabilitation to reestablish metabolic equilibrium. Translational evidence demonstrates metformin‐induced AMPK activation decreases inflammatory mediators [193], puerarin‐mediated SIRT1 elevation preserves renal function [194], and PGC‐1α overexpression confers hypoxia resistance in renal epithelial cells [195]. In summary, the AMPK/SIRT1/PGC‐1α pathway serves as a central hub integrating multiple pathological processes in CKD (inflammation, fibrosis, and metabolic dysregulation), making it a highly promising therapeutic target. By orchestrating a cross‐scale regulatory network connecting inflammation, fibrosis, and energy metabolism, targeted activation of this pathway may restore renal microenvironmental homeostasis, offering a novel approach for developing precision intervention strategies in CKD.

#### 4.5.2 Chronic Obstructive Pulmonary Disease (COPD)

Chronic obstructive pulmonary disease (COPD) involves ongoing airway inflammation, an imbalance between protein‐digesting enzymes and their inhibitors, and oxidative damage. These problems worsen due to a malfunctioning AMPK/SIRT1/PGC‐1α pathway [196, 197]. This faulty signaling speeds up COPD through three connected problems. First, cigarette smoke causes oxidative damage while lowering SIRT1 levels in both COPD patients and lab animals. When SIRT1 can't properly modify FOXO3, it reduces protective antioxidant enzymes [198, 199, 200]. Damaged DNA markers in COPD lungs directly link to poor SIRT1 function [173], proving its importance. Second, weak AMPK activity in lung immune cells allows harmful inflammation signals to spread, increasing damaging chemicals like TNF‐α/IL‐8 [201]. Mice lacking AMPKα1 develop worse lung inflammation and emphysema when exposed to smoke [202]. Third, low SIRT1 levels overactivate an enzyme called p300, which disrupts the balance between tissue‐destroying MMP‐9 and its inhibitor α1‐antitrypsin, leading to lung damage [203, 204]. However, treatments targeting this pathway can help. The SIRT1‐FOXO3 connection boosts antioxidants, AMPK blocks inflammation signals, and SIRT1 rebalances destructive enzymes. Emerging evidence highlights multiple pharmacologic agents with distinct mechanisms. Resveratrol potentiates SIRT1‐mediated activation of endogenous antioxidant systems [205], whereas quercetin restores AMPK‐dependent cytoprotective pathways in alveolar epithelium [206]. Nicotinamide mononucleotide promotes macrophage phenotypic switching to tissue‐reparative states [207]. Beyond these effects, SIRT1 enzymatically rebalances the MMP‐9/TIMP‐1 axis via targeted protein modifications [203, 204]. These findings highlight how the AMPK/SIRT1/PGC‐1α pathway controls COPD's key problems and could lead to better treatments. In conclusion, dysfunction of the AMPK/SIRT1/PGC‐1α pathway represents a pivotal mechanism in COPD pathogenesis (Table 1). Novel therapeutic opportunities emerge from targeted activation strategies that simultaneously engage antioxidant defenses, anti‐inflammatory pathways and proteostasis restoration mechanisms. Elucidating the pathway's dynamic regulatory network within the pulmonary immunometabolic microenvironment, coupled with developing tissue‐specific delivery systems to enhance targeting efficacy, will facilitate a paradigm shift from symptomatic management to disease modification in COPD treatment.

## 5 Therapeutic Strategies Targeting the AMPK/SIRT1/PGC‐1α Signaling Pathway

### 5.1 Small‐Molecule Drug Development

#### 5.1.1 AMPK Activators

**FIGURE 3:**
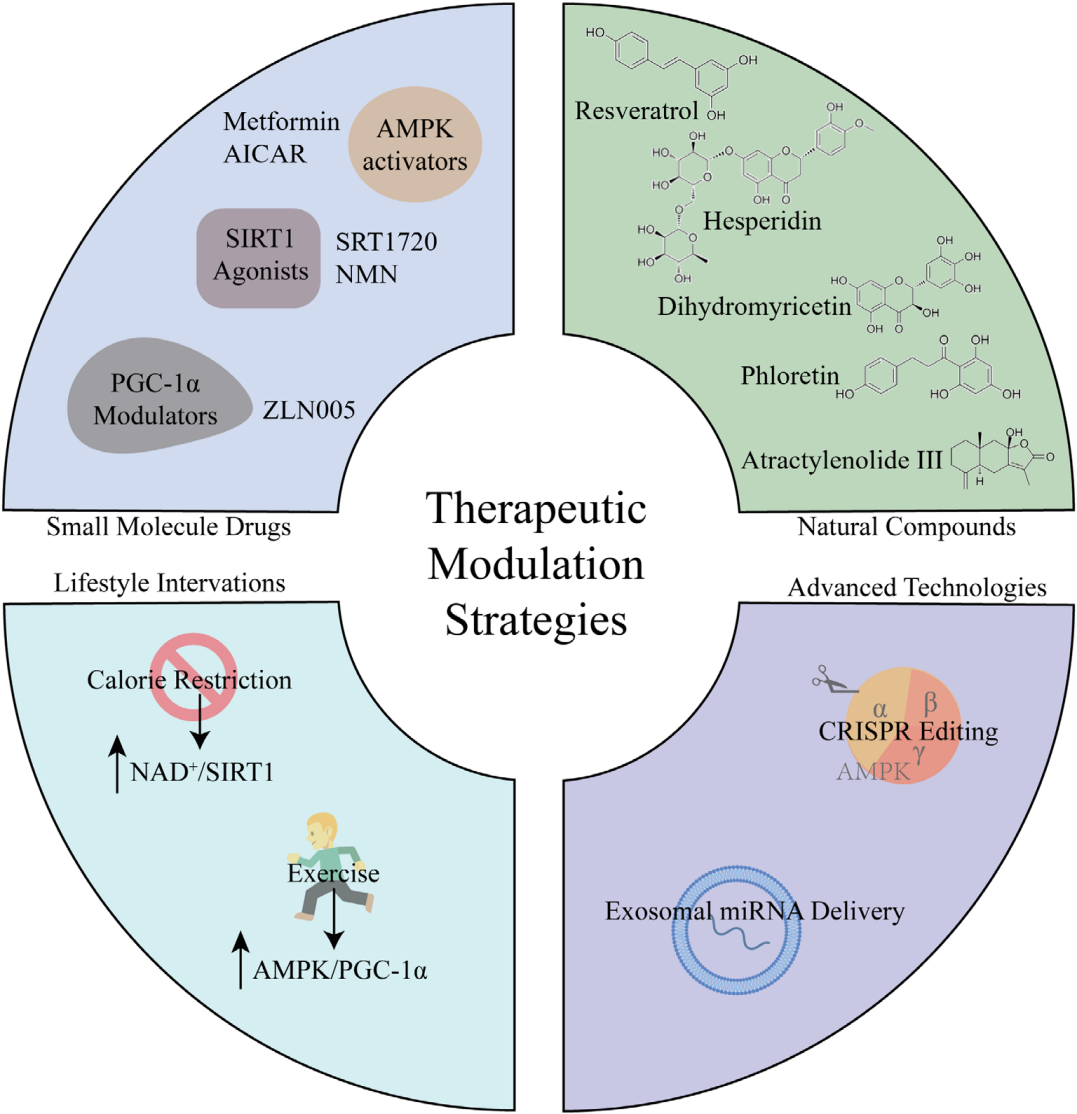
Therapeutic strategies targeting the AMPK/SIRT1/PGC‐1α signaling pathway. Small molecule drugs targeting this pathway include AMPK Activators, SIRT1 Activators and PGC‐1α Modulators; Natural Compounds include Resveratrol, Hesperidin, Dihydromyricetin, Phloretin, Atractylenolide III, etc. Caloric restriction and Exercise Intervention are also effective. In addition, Gene therapy and exosome therapy are also being explored.

Researchers have made significant advances in developing AMPK activators, with metformin and AICAR standing out as key examples showing protective effects across multiple organs (Figure 3). Metformin works by blocking mitochondrial complex I, which activates AMPK through phosphorylation. Metformin's mechanism provides multiple therapeutic advantages. It enhances metabolic function by promoting glucose uptake in liver, muscle and adipose tissue while suppressing hepatic gluconeogenesis, establishing its position as a first‐line diabetes therapy [208]. Additionally, it confers cardiorenal protection through AMPK‐mediated reduction of inflammatory responses, restoration of autophagic flux and inhibition of apoptosis [209]. In diabetic kidney disease specifically, metformin activates the AMPK/SIRT1 axis to mitigate oxidative damage and prevent excessive autophagy [210]. Metformin demonstrates extended therapeutic potential in oncology and musculoskeletal health. It induces cancer cell apoptosis via AMPK/SIRT1‐dependent degradation of NF‐κB p65 [211], while protecting against osteoarthritis by enhancing chondrocyte autophagy, inhibiting apoptosis and regulating cholesterol metabolism [212]. Regarding hepatic and vascular systems, metformin selectively inhibits PGC‐1α‐mediated hepatic gluconeogenesis [213] and enhances telomerase activity through AMPK/PGC‐1α phosphorylation to prevent arterial stiffening [214]. The AMP‐mimetic AICAR operates through unique pharmacologic mechanisms as a direct AMPK agonist. Its metabolic reprogramming capabilities include potentiation of fatty acid β‐oxidation, GLUT4‐mediated glucose uptake and glycolytic flux [215]. AICAR's organoprotective properties manifest differentially across tissues: it reduces renal oxidative stress and inflammation while maintaining SIRT1 homeostasis during ischemic injury [216]; reverses neurobehavioral abnormalities and AMPK/SIRT1 deficits in chronic stress models [217]; and ameliorates obesity‐induced insulin resistance through SIRT1‐dependent pathways [218]. Muscle‐specific responses reveal time‐dependent PGC‐1α regulation, where transient AICAR exposure upregulates PGC‐1α and glycogen synthesis, but prolonged treatment elicits opposite effects [213]. These activators provide novel therapeutic strategies for metabolic disorders, organ damage and degenerative diseases through precise modulation of AMPK networks. Future research should focus on developing tissue‐selective activators and elucidating spatiotemporal dynamics in microenvironmental contexts, aiming to overcome off‐target limitations and achieve precise metabolic reprogramming.

#### 5.1.2 SIRT1 Activators

SIRT1 activators show great promise for treating age‐related diseases, with SRT1720 and nicotinamide mononucleotide (NMN) standing out as particularly effective options that work in different ways (Figure 3). SRT1720 selectively activates SIRT1 to provide cellular protection through two primary mechanisms. First, it enhances PGC‐1α transcriptional activity to mitigate toxin‐induced cellular damage, as demonstrated in paraquat exposure models [219]. Second, it ameliorates vascular dysfunction in atherosclerosis by restoring mitochondrial homeostasis and suppressing proinflammatory signaling pathways [220]. Meanwhile, NMN works differently as it helps produce NAD^+^, which then activates SIRT1's protein‐modifying abilities. This gives NMN its brain‐protecting effects, seen in its ability to reduce inflammation and oxidative stress in the memory centers of mice with severe infections [221]. NMN also helps maintain healthy metabolism in diabetes by preventing the usual drop in SIRT1 and PGC‐1α activity, using its protein‐modifying power to shield against high blood sugar damage [222]. These two compounds collectively illustrate how SIRT1 activation, whether achieved directly through SRT1720 or indirectly via NMN‐mediated NAD^+^ elevation, can offer substantial therapeutic benefits for multiple age‐related conditions. Considering both the limited availability of SIRT1‐targeting drugs and its crucial role in lifespan regulation, researchers must prioritize two critical areas. First, they should explore the therapeutic potential of SIRT1 activators for neurodegenerative diseases such as AD and age‐related metabolic disorders including T2DM. Second, they need to clarify how tissue‐specific deacetylation networks interact with epigenetic regulation. The development of blood–brain barrier‐permeable SIRT1 allosteric activators, combined with deciphering their tissue‐specific epigenetic remodeling mechanisms, may overcome current therapeutic bottlenecks in age‐related diseases. This approach could ultimately catalyze a paradigm shift from lifespan extension to healthspan prolongation.

#### 5.1.3 PGC‐1α Modulators

The PGC‐1α agonist ZLN005 shows protective effects across multiple organs through three key mechanisms, making it a promising new treatment for metabolic and degenerative diseases (Figure 3). For heart protection, ZLN005 reduces oxidative damage caused by chemotherapy drugs, improves cellular antioxidant defenses, prevents harmful tissue changes, and slows the progression of heart muscle disease [223]. In the nervous system, it activates mitochondrial protein production, maintains proper energy levels in neurons, and helps brain cells survive under stressful conditions [224]. The compound also protects against damage caused by restored blood flow after oxygen deprivation in organs like the heart, brain and kidneys by boosting mitochondrial growth and reducing harmful reactive oxygen species [225]. By directly targeting the central mitochondrial energy production system, ZLN005 offers an innovative treatment approach that addresses the root causes of these disorders rather than just their symptoms. This compound offers innovative therapeutic strategies for metabolic and degenerative diseases by directly modulating core mitochondrial energy metabolism pathways. Future research should focus on developing tissue‐specific allosteric activators coupled with spatiotemporally precise drug delivery systems to decipher dynamic metabolic reprogramming effects. Addressing current organ‐specific delivery challenges could enable a fundamental transformation in treatment approaches, moving beyond symptomatic management to achieve genuine tissue regeneration.

### 5.2 Natural Compounds and Phytochemicals

Natural compounds show great promise for treating various diseases by precisely adjusting the AMPK/SIRT1/PGC‐1α pathway (Figure 3), providing new approaches for metabolic disorders, brain diseases, and organ damage. These plant‐based molecules work through different but complementary ways. Stilbenoids like resveratrol increase NAD^+^ levels to activate SIRT1, which then boosts AMPK activity and modifies PGC‐1α to repair mitochondria [226, 227]. This helps protect blood vessels by reducing cell death in eye capillaries caused by high sugar [228], improves metabolism by helping fat cells take in glucose better [229], protects nerves by enhancing cell cleanup in spinal cord injuries [230] and reducing harmful protein buildup in Alzheimer's models [231], and even changes muscle fiber types [232]. Flavonoids work similarly—hesperidin activates AMPK/SIRT1 to help with liver inflammation [233], regulate mitochondria in fatty liver disease, and improve diabetes‐related problems [234, 235]. Quercetin stops harmful inflammation by blocking specific immune cell death pathways and protects nerve cells in ALS [236, 237]. Dihydromyricetin adjusts sugar and fat metabolism [238]. Phloretin ameliorates diabetic vascular endothelial dysfunction through the GLUT1 inhibition–AMPK activation–PGC‐1α upregulation cascade [239], while mulberry flavonoids activate the AMPK‐PGC‐1α axis to enhance skeletal muscle glucose uptake and mitochondrial function, thereby reversing insulin resistance [240]. Notably, the terpenoid compound atractylenolide III exerts protective effects against non‐alcoholic fatty liver disease by activating the AMPK/SIRT1 pathway to suppress lipid accumulation and oxidative stress [241]. These compounds demonstrate multidimensional metabolic remodeling and cytoprotective activities through multi‐target modulation of the AMPK/SIRT1/PGC‐1α network. Future research should focus on developing organ‐specific delivery systems to enhance targeting efficiency, employing single‐cell metabolomics to decipher spatiotemporal regulatory mechanisms, and implementing AI‐driven structure–activity relationship optimization. Such advances will facilitate the paradigm shift of natural compounds from empirical medicine to precise metabolic intervention.

### 5.3 Non‐Pharmacological Intervention Strategies

#### 5.3.1 Caloric Restriction (CR)

Caloric restriction (CR), which means eating fewer calories while still getting essential nutrients, is a proven way to improve health and extend lifespan without genetic changes (Figure 3) [242]. It works by carefully adjusting cell cleanup processes, nutrient signals, and key energy pathways. CR helps maintain metabolic balance by reducing cellular stress, lowering blood sugar, and improving insulin response [243]. It activates energy‐sensing pathways by increasing the NAD^+^/NADH ratio to boost SIRT1 activity, while also enhancing AMPK activation and promoting PGC‐1α‐driven mitochondrial growth [244, 245]. For heart health, CR improves energy use in heart cells through the AMPK/SIRT1/PGC‐1α system and makes the heart more resistant to stress [246]. These combined effects boost mitochondrial production, improve metabolic balance, and reduce cellular stress, effectively slowing aging while improving metabolic health and heart function. Important next steps include using advanced testing methods to determine personal calorie restriction responses, creating detailed biomarker profiles based on AMPK/SIRT1/PGC‐1α pathway activity, developing timed eating plans that copy calorie restriction benefits, and applying AI technology to create personalized anti‐aging treatments. This approach will move us from general diet changes to customized health plans targeting specific biological mechanisms, transforming how we prevent age‐related diseases. Research should especially examine how different tissues and genetic backgrounds respond to identify the most effective treatments with the fewest side effects, helping create tailored solutions for aging populations.

#### 5.3.2 Exercise Intervention

Exercise triggers metabolic changes by activating the AMPK/SIRT1/PGC‐1α pathway through three key steps (Figure 3). First, during the energy stress phase, muscles quickly take up glucose while breaking down glycogen and making new glucose, leading to ATP depletion and an increased AMP/ATP ratio that activates AMPK [247, 248]. Second, this AMPK activation boosts PGC‐1α production, remodeling mitochondria to burn more fat [249]. Third, exercise protects the brain by improving cell cleanup through AMPK‐SIRT1 signaling [250] and reducing brain inflammation in aging animals [251]. Interestingly, the plant compound DMC enhances these exercise benefits by strengthening AMPK/SIRT1/PGC‐1α signaling in muscles and fat, improving metabolism, reducing inflammation and maintaining energy balance [252]. Collectively, these findings highlight the AMPK/SIRT1/PGC‐1α pathway as a central orchestrator of exercise‐induced metabolic adaptation, bridging acute energy stress with long‐term tissue remodeling, while pharmacological mimetics like DMC may synergize with physical activity to optimize metabolic health.

### 5.4 Gene Therapy and Exosome Therapy

CRISPR/Cas9 gene editing has helped scientists understand AMPK's key roles in metabolism and cancer treatment (Figure 3). By editing AMPK subunits, researchers found that restoring AMPKα1 in modified cells fixes problems with cellular cleanup processes [253]. In cancer studies, removing both AMPK subunits showed it controls energy production in brain tumors through PGC‐1α activation—similar to adding extra PGC‐1α [254]. When testing a potential cancer drug, deleting AMPK made the treatment ineffective, proving AMPK is essential for its anti‐cancer action [255]. CRISPR/Cas9‐mediated targeted editing of AMPK subunits has elucidated its pivotal role in regulating metabolic disorders, while demonstrating that intact AMPK functionality is essential for the antitumor effects of pharmacological activators. These findings establish a molecular foundation for developing precision cancer therapeutics targeting the AMPK‐PGC‐1α axis.

Exosomes are tiny communication bubbles released by cells that play important roles in both health and disease, serving as carriers for potential disease markers and showing treatment potential for various conditions [256]. These natural delivery systems demonstrate significant potential for AMPK‐targeted therapies in various applications (Figure 3). In metabolic disorders, umbilical cord stem cell‐derived exosomes activate AMPK to facilitate muscle recovery in diabetes [257], while Lycium berry nanoparticles enhance muscle performance by elevating AMPK/SIRT1/PGC‐1α pathway activity [258]. In tissue repair, exosomes combined with the diabetes drug metformin use the AMPK/SIRT1 pathway to rebuild damaged liver mitochondria [259]. Researchers have also discovered that harmful exosomes from certain immune cells can worsen lung blood pressure by blocking AMPK/SIRT1 signaling, revealing new disease mechanisms [260]. Looking forward, the integration of CRISPR's precision gene‐editing capability with the targeted delivery potential of exosomes represents a promising frontier. However, the clinical translation of these advanced technologies critically depends on overcoming major challenges in delivery efficiency and targeting specificity. Successfully harnessing this dual‐approach strategy could ultimately reprogram cellular metabolism and open new therapeutic avenues for metabolic disorders and other chronic diseases.

## 6 Current Challenges and Future Perspectives

The AMPK/SIRT1/PGC‐1α signaling pathway functions as the core regulator of cellular energy balance, creating an integrated network that connects metabolic control, aging processes and stress responses through its three‐component system of energy sensing (AMPK), epigenetic regulation (SIRT1) and transcriptional control (PGC‐1α). This review systematically examines the pathway's molecular mechanisms, pathological implications and therapeutic potential.

As the primary energy sensor within this network, the AMPK exhibits a profound and context‐dependent “double‐edged sword” effect. On one hand, AMPK acts as a potent tumor suppressor. It can phosphorylate and activate the deubiquitinase BAP1, leading to stabilization of the tumor suppressor pVHL and inhibiting tumor progression [261]. Furthermore, AMPK serves as a negative regulator of the Warburg effect and suppresses tumor growth in vivo by inhibiting aerobic glycolysis and mTORC1 signaling [262]. This anti‐tumor capacity is leveraged therapeutically, as evidenced by the AMPK agonist AICAR inhibiting glioblastoma growth through suppression of lipogenesis [263]. Conversely, AMPK can also promote tumor survival under metabolic stress. In nutrient‐deprived conditions common in solid tumors, AMPK maintains NADPH homeostasis by inhibiting acetyl‐CoA carboxylases (ACC1/ACC2), thereby protecting cancer cells from oxidative stress and death and facilitating their survival [264]. This pro‐survival role is further highlighted by recent findings that the metabolite α‐ketoglutarate is required for AMPK protein synthesis via a TET‐YBX1 axis; cancer cells lacking this axis fail to produce AMPK and become sensitized to energy stress‐induced death, underscoring AMPK's critical role in stress adaptation [265]. Additionally, in glioblastoma, the TBL2 gene promotes tumor progression by inducing autophagy through the AMPK/mTOR pathway, demonstrating how AMPK‐mediated survival processes can be co‐opted for tumor aggression [266]. In conclusion, AMPK exerts a dual role in cancer. It primarily functions as a gatekeeper against tumorigenesis by restraining anabolic growth, yet in established tumors facing metabolic challenges, its activation can paradoxically support cancer cell adaptation and survival. This intricate duality necessitates context‐specific therapeutic strategies, either activating AMPK for prevention or targeting it to undermine tumor resilience in advanced disease.

The α subunit of AMPK exists in two isoforms, α1 and α2, which are widely expressed across various tissues. Accumulating evidence highlights their distinct functional roles—not only across different tissues but also within specific cell types of the same tissue. In skeletal muscle, AMPKα2 is essential for regulating exercise‐induced glucose uptake. Upon physical activity, AMPKα2 is rapidly activated and translocates to the nucleus, where it modulates transcriptional programs to meet cellular energy demands [267]. In contrast, AMPKα1 plays a critical role in restraining excessive skeletal muscle growth during hypertrophy [268]. In the heart, suppression of AMPKα2 leads to pathological cardiac hypertrophy [269], whereas basal AMPKα1 activity is more closely associated with maintaining normal mitochondrial function and autophagic processes [270], indicating that both isoforms act synergistically to preserve cardiac metabolic homeostasis and stress resilience. In the brain, AMPK complexes containing the α2 subunit function as specific sensors of intracellular amino acid abundance, regulating protein synthesis; dysregulation of this pathway has been linked to the pathogenesis of Alzheimer's disease (AD) [271]. Conversely, impaired AMPKα1 activity is more strongly associated with cognitive deficits and synaptic dysfunction [272]. These findings underscore significant tissue‐ and context‐specific functional divergence between AMPKα1 and α2, driven by differences in expression patterns, subcellular localization, upstream activating signals, and substrate selectivity. This isoform‐specific functional specialization not only elucidates the precise regulatory capacity of AMPK in systemic metabolic integration but also provides a crucial foundation for developing targeted and safer therapeutic interventions for metabolic and neurodegenerative disorders.

Despite substantial progress in AMPK/SIRT1/PGC‐1α pathway research, several critical challenges persist (Table 2). First, tissue‐specific regulatory mechanisms remain poorly understood, particularly regarding AMPK isoform expression patterns, SIRT1 substrate profiles, and PGC‐1α interactomes across different organs. This knowledge gap requires single‐cell multi‐omics approaches to build comprehensive tissue atlases and investigate microenvironmental influences like blood–brain barrier effects on pathway activity. Second, current technologies for spatiotemporal monitoring are inadequate for real‐time tracking of AMP/ATP ratios, NAD^+^ levels, and protein–protein interactions in vivo. Developing genetically encoded biosensors such as AMPK activity reporter mice combined with super‐resolution imaging could address this limitation. Third, quantitative models of complex feedback networks are lacking, hindering our understanding of crosstalk with PI3K/AKT, NF‐κB, and mTOR pathways. Computational modeling is needed to quantify regulatory node weights, including NAD^+^‐dependent activation thresholds for AMPK‐mediated SIRT1 activation. Finally, translational challenges persist, including delivery efficiency issues that demand targeted liposomes for improved organ accumulation, off‐target risks requiring β1‐specific allosteric modulators, and a clinical translation gap that could benefit from AI‐driven structure–activity optimization to accelerate the development of lead compounds like quercetin derivatives.

**TABLE 2:** Current challenges and future perspectives.

Challenges	Solutions
Tissue‐specific differences Divergent SIRT1 substrate profiles (e.g., brain vs. muscle)	Single‐cell multi‐omics Tissue‐specific molecular atlas construction
2 Dynamic monitoring gaps Real‐time NAD^+^ detection limitations	Genetically encoded biosensors AMPK fluorescent reporter mice NAD^+^‐sensing nanoprobes
3 Delivery efficiency barriers Poor blood–brain barrier penetration	Targeted exosome systems Brain‐homing peptide‐modified vesicles miRNA‐loaded exosomes

To overcome these challenges, future studies should combine three innovative approaches to transform current paradigms. First, researchers should create multimodal intervention platforms merging CRISPR activation with engineered exosomes, enabling dual gene‐editing and vesicle delivery for tissue‐specific pathway activation. Second, scientists need to build personalized digital twin models using patient multi‐omics data to predict drug responses. Third, the field should develop cross‐scale therapies that integrate pathway modulation with neurovascular protection and immunometabolic reprogramming to address complex conditions like diabetes–Alzheimer's comorbidity. By elucidating spatiotemporal regulation, developing advanced delivery systems, and integrating AI with systems biology, research on the AMPK/SIRT1/PGC‐1αwill progress from basic mechanistic studies to precise metabolic control, ultimately achieving the goal of multidimensional therapy via a single pathway.

## Funding

This study was supported by the National Natural Science Foundation of China (Nos. 32130060, 82401633), the Natural Science Foundation of Jiangsu Province (No. BK20232023), the Natural Science Research Projects in Universities of Jiangsu Province (No. 24KJA310007, No. 24KJB310013), and the Suzhou Science and Technology Development Program (No. SYW2024048).

## Conflicts of Interest

The authors declare no conflicts of interest.

## Data Availability

The authors have nothing to report.
